# Temporal patterning in neural progenitors: from *Drosophila* development to childhood cancers

**DOI:** 10.1242/dmm.044883

**Published:** 2020-07-22

**Authors:** Cédric Maurange

**Affiliations:** Aix Marseille University, CNRS, IBDM, Equipe Labellisée LIGUE Contre le Cancer, Marseille 13009, France

**Keywords:** *Drosophila*, Pediatric cancer, Neural stem cell, Temporal patterning, Medulloblastoma

## Abstract

The developing central nervous system (CNS) is particularly prone to malignant transformation, but the underlying mechanisms remain unresolved. However, periods of tumor susceptibility appear to correlate with windows of increased proliferation, which are often observed during embryonic and fetal stages and reflect stereotypical changes in the proliferative properties of neural progenitors. The temporal mechanisms underlying these proliferation patterns are still unclear in mammals. In *Drosophila*, two decades of work have revealed a network of sequentially expressed transcription factors and RNA-binding proteins that compose a neural progenitor-intrinsic temporal patterning system. Temporal patterning controls both the identity of the post-mitotic progeny of neural progenitors, according to the order in which they arose, and the proliferative properties of neural progenitors along development. In addition, in *Drosophila*, temporal patterning delineates early windows of cancer susceptibility and is aberrantly regulated in developmental tumors to govern cellular hierarchy as well as the metabolic and proliferative heterogeneity of tumor cells. Whereas recent studies have shown that similar genetic programs unfold during both fetal development and pediatric brain tumors, I discuss, in this Review, how the concept of temporal patterning that was pioneered in *Drosophila* could help to understand the mechanisms of initiation and progression of CNS tumors in children.

## Abstract (precis)

**Summary:** Temporal patterning is the mechanism by which neural progenitors change their competence and proliferative properties during development. Recent data suggest that co-opted temporal patterning could govern tumor growth in neural cancers with developmental origins.

## Introduction: the developmental origins of CNS tumors in children

It is now well accepted that the etiology of adult and childhood cancers underlies different mechanisms and principles. Adult cancer is mainly a disease of aging and incidence peaks after 65 years of age, with about one-fifth of the population expecting to develop cancer in their adult lives. The progression of cancers in adults relies on the progressive accumulation of genomic alterations (single-nucleotide variants, copy number alterations or structural rearrangements of the chromosome) and is an evolving process that can span several years. In contrast, cancers in children (0-15 years of age), also known as pediatric cancers, are much rarer, but develop rapidly, despite exhibiting a much lower mutational burden than adult cancers (Gröbner et al., 2018). Interestingly, the spectrum of cancers in children differs from the spectrum of cancers in adults. Carcinomas arising from epithelial tissues are largely dominant in adults (Bray et al., 2018), but uncommon in children. In contrast, cancers of the central nervous system (CNS) are over-represented in children (25% of all cancers in children versus 2% in adults) (Arora et al., 2009). There are now strong indications that the important proportion of CNS cancers in children could be due to an increased malignant susceptibility of neural tissues during embryonic and/or fetal development.

Most pediatric CNS cancers are indeed initiated during pregnancy while the CNS is being built. For example, the different types of medulloblastoma, the most frequent CNS tumors in children, appear to all originate from different fetal precursors of the cerebellum and dorsal brain stem (Gibson et al., 2010; Vladoiu et al., 2019), while fetal glial-committed progenitors could be at the root of pediatric high-grade gliomas (Jessa et al., 2019; Pathania et al., 2017). Strikingly, retinoblastoma and atypical teratoid rhabdoid tumors (ATRTs) are sometimes diagnosed at birth (Puisieux et al., 2018). Work on human retinal progenitors has demonstrated that retinoblastomas originate from cone photoreceptor precursors produced during fetal development (Xu et al., 2014), whereas cells of the early neural crest, an embryonic structure lying on the dorsal side of the neural tube, may be the cells of origin of some ATRTs (Vitte et al., 2017). Interestingly, retinoblastomas and ATRTs are induced by the inactivation of a single gene: *RB1*, coding for retinoblastoma-associated protein, and *SMARCB1*, coding for SWI/SNF-related matrix-associated actin-dependent regulator of chromatin subfamily B member 1, respectively (Puisieux et al., 2018). Despite such simple mutational landscapes, ATRTs and retinoblastomas can be very aggressive, and there is so far no effective treatment for ATRTs, leading to poor prognosis (Frühwald et al., 2016). Taken together, these data lead to the hypothesis that neural cells or progenitors, present during embryonic and fetal development in the different regions of the developing CNS, exhibit transient periods of malignant susceptibility during which they may be prone to initiate tumorigenesis upon specific genomic alterations.

So far, it is unclear what defines a malignant susceptibility state in a specific neural progenitor at a given time during development. Is it an intrinsic and transient property of neural progenitors determined by the progression of an intrinsic genetic program? Is it due to a specific and transient microenvironmental context producing specific cocktails of growth factors? Which gene networks in cells of origin support oncogenic growth during early development? Why and how do they become aberrantly expressed in neural progenitors to initiate tumor growth?

These questions remain largely unanswered because neural progenitors are rare, diverse and represent transient populations of cells, which are, therefore, difficult to access and investigate at high resolution. Understanding the developmental mechanisms controlling neural progenitor proliferation in the embryo and fetus is, however, key to deciphering the mechanisms supporting the initiation and growth of tumors with early developmental origins (Azzarelli et al., 2018; Jessa et al., 2019).

The past few years have seen the advent of revolutionary technologies that allow us to capture and manipulate the transient nature of many neural precursor states during mammalian development. In parallel, decades of meticulous and persistent studies on simple model organisms, such as *Drosophila*, have led to an unmatched understanding of the genetic mechanisms regulating patterns of neural progenitor proliferation throughout the course of development. Here, I summarize how the discovery of a dynamic network of genes, known as temporal patterning, in *Drosophila* neural progenitors, provides concepts to decipher the links between development and pediatric CNS cancers. I also examine recent single-cell transcriptomic studies in mammals in light of these concepts.

## Temporal patterning: a versatile system to coordinate cell fates and numbers in neural lineages during development

**Fig. 1.**
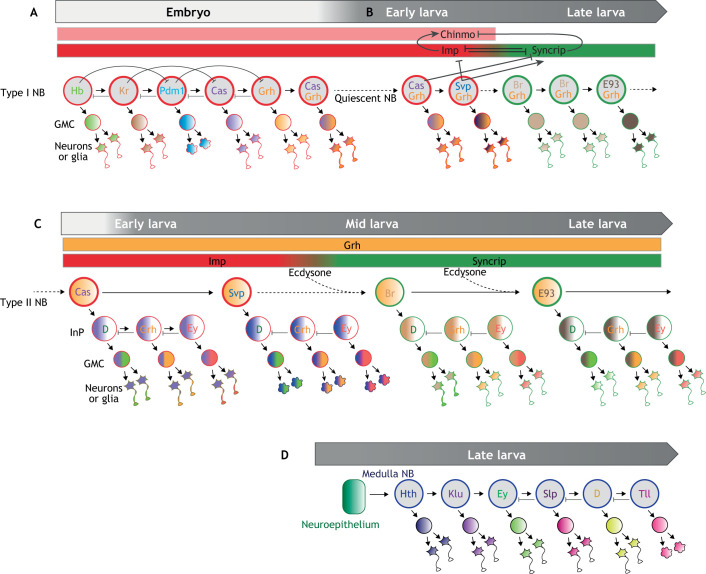
**Temporal patterning systems in *Drosophila* NBs.** (A) Type I NBs divide asymmetrically to self-renew and generate GMCs. The latter divide usually once, giving rise to two post-mitotic progeny: neurons or glia. Embryonic NBs in the ventral nerve cord sequentially express a series of ‘temporal’ transcription factors (tTFs) (Hb→Kr→Pdm1→Cas→Grh). Progression throughout the sequence is driven by transcriptional cross-regulatory interactions between tTFs. Sequential expression of tTFs ensures the production of daughter cells with different fates by the same NB. Co-expression of two temporal factors (e.g. Cas and Grh) can create novel temporal windows. (B) A subset of NBs enters quiescence by the end of embryogenesis and resumes divisions in early larvae. Cas and Svp are sequentially expressed to promote a switch in the expression of Imp and Syncrip, thereby creating two large post-embryonic temporal windows (Imp^+^ and Syncrip^+^, respectively). The late Syncrip^+^ window is subdivided into two subtemporal windows marked by the expression of Br and E93. (C) Type II NBs in the central brain generate intermediate progenitors (InPs) that divide a few times and exhibit their own temporal patterning system (D→Grh→Ey). Concomitant progression of two distinct temporal patterning systems in larval NBs and their InPs increases neural diversity in large lineages. Ecdysone signaling facilitates the Imp-to-Syncrip transition and promotes the expression of E93 (and possibly Br). (D) Medulla NBs are converted from a neuroepithelium in the larval optic lobes. They express another series of tTFs (Hth→Klu→Ey→Slp→D→Tll) to specify the identity of their progeny. GMC, ganglion mother cell; InP, intermediate progenitor; NB, neuroblast.

Over the past 30 years, *Drosophila* neural progenitors, called neuroblasts (NBs), have become a powerful model to explore neural stem cell biology (Homem and Knoblich, 2012). Throughout development, NBs divide asymmetrically to self-renew while generating daughter cells with a more restricted cycling potential. Type I NBs (most NBs in the *Drosophila* CNS), generate daughter cells known as ganglion mother cells (GMCs). GMCs usually divide once, giving rise to two post-mitotic progeny: neurons or glia (Fig. 1A). A small subset of NBs producing larger lineages is known as type II NBs. They generate daughter cells, called intermediate progenitors (InPs), themselves able to undergo a few rounds of asymmetric divisions (Fig. 1C). About two decades ago, it was observed that type I NBs in the ventral nerve cord of *Drosophila* embryos express a sequence of transcription factors able to endow their differentiated progeny with different identities (Isshiki et al., 2001; Kambadur et al., 1998). The concept of temporal patterning was born. Since then, temporal patterning has been extended to NBs in other regions of *Drosophila* CNS, which display different sequences of temporal transcription factors (tTFs) (Box 1).

**Box 1. Temporal patterning in *Drosophila* neuroblasts**

Temporal patterning is the mechanism by which stem cells or progenitors change their competence as development progresses in order to generate different types of differentiated progeny. This concept has been pioneered in *Drosophila*, with the observation that embryonic neuroblasts (NBs) found in the ventral nerve cord (type I NBs) sequentially express a series of transcription factors [Hunchback (Hb)→Kruppel (Kr)→Pou-domain protein 1 (Pdm1; also known as Nub)→Castor (Cas)→Grainyhead (Grh)] as they undergo successive asymmetric divisions (Brody and Odenwald, 2002; Isshiki et al., 2001; Kambadur et al., 1998). The so-called ‘temporal transcription factors’ (tTFs) are inherited by each newborn progeny, thereby specifying their fate according to the order in which they arose. This mechanism leads to stereotypical sequences of glial and neuronal identities being generated by each NB (Fig. 1A). The combination of temporal patterning with spatial patterning, that specifies NB identity according to their position along the different body axes (e.g. antero-posterior, dorso-ventral), provides a system to leverage the diversity of neurons and glia produced by a small population of NBs (Erclik et al., 2017; Karlsson et al., 2010). A subset of embryonic NBs persists to larval stages and sequentially expresses Cas and another tTF, Seven up (Svp) (Benito-Sipos et al., 2011; Kanai et al., 2005; Maurange et al., 2008). Svp expression triggers a switch in the expression of two antagonistic RNA-binding proteins, IGF-II mRNA-binding protein (Imp) and Syncrip (Syp) (Fig. 1B). Imp and Syncrip post-transcriptionally regulate multiple genes, including the transcription factor Chronologically inappropriate morphogenesis (Chinmo), to switch the fate of the progeny produced by late larval NBs (Liu et al., 2015; Maurange et al., 2008; Ren et al., 2017). Syncrip^+^ NBs also sequentially express the transcription factors Broad (Br) and Ecdysone-induced protein 93F (E93) that provides novel subtemporal windows in late larval NBs (Fig. 1B). Type II NBs found in the central brain produce InPs that divide a small number of times and transit throughout their own temporal sequence [Dichaete (D)→Grainyhead (Grh)→Eyeless (Ey)] (Bayraktar and Doe, 2013) (Fig. 1C). This system combined with the Imp/Syncrip temporal windows in larval NBs provides ‘combinatorial’ temporal patterning to further expand neuronal diversity in large lineages (Fig. 1C) (Ren et al., 2017). Another series of tTFs [Homothorax (Hth)→Kumpfuss (Klu)→Eyeless (Ey)→Sloppy paired 1/2 (Slp)→Dichaete (D)→Tailless (Tll)] has been uncovered in medulla NBs that are converted from a neuroepithelium in the optic lobe region of the larval brain (Fig. 1D) (Li et al., 2013). In ventral nerve cord and medulla NBs, as well as in intermediate progenitors, progression of temporal patterning is mainly driven by cross-regulatory interactions between tTFs (sometimes involving feed-forward loops and chromatin-remodeling factors), which ensures discrete temporal transitions and unidirectionality (Fig. 1) (Abdusselamoglu et al., 2019; Averbukh et al., 2018; Grosskortenhaus et al., 2005). Some temporal transitions appear to require cell-cycle progression or high levels of oxidative phosphorylation (Grosskortenhaus et al., 2005; van den Ameele and Brand, 2019). Although temporal patterning progression in most NBs appears to be mainly intrinsically driven, external cues, such as the steroid hormone ecdysone, may also facilitate temporal transitions, especially in type II NBs (Fig. 1C) (Dillard et al., 2018; Syed et al., 2017). Temporal patterning is also used to modulate the proliferative properties of stem cells and their daughter cells, ensuring stereotypical patterns of proliferation along lineage progression (see main text).

**Fig. 2.**
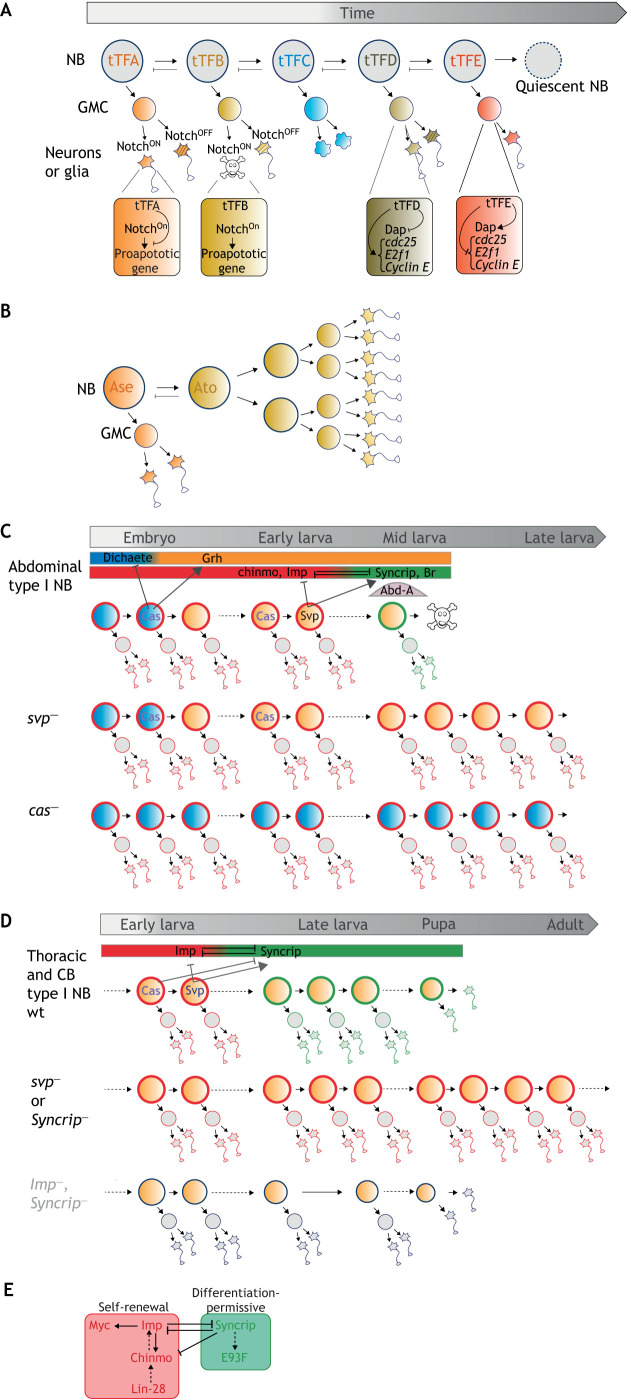
**Stereotyped regulation of neural progenitor proliferation by temporal patterning.** (A) Temporal regulation of daughter cell divisions or apoptosis. Notch signaling in GMCs gives rise to binary cell fate decisions in the two post-mitotic progeny. The Notch^ON^ state can trigger activation of pro-apoptotic genes leading to the elimination of one of the progeny. Some tTFs have the ability to repress Notch^ON^-mediated apoptosis. tTFs can also block the division of GMCs or promote NB quiescence by activating Dap and silencing cell-cycle genes (*string*/*cdc25*, *E2f1*, *Cyclin E*), leading to their terminal differentiation without division. (B) Temporal regulation of an asymmetric-to-symmetric division switch. tTF progression can also promote a switch in the NB mode of division. In the larval optic lobe, a subset of NBs undergoes an asymmetric-to-symmetric division switch triggered by the sequential expression of Ase and Ato. (C) Temporal regulation of NB apoptosis. Permanent transcriptional switches induced by tTFs (embryonic expression of *cas* and larval expression of *svp*) schedule the competence for NBs to undergo apoptosis upon expression of *abd-A.* (D) Temporal regulation of NB self-renewal. Sequential expression of *cas* and *svp* promotes the Imp-to-Syncrip switch during mid-larval stages. NBs that fail to undergo the Imp-to-Syncrip switch do not stop dividing during pupal stages and continue dividing in adults. NBs lacking both Imp and Syncrip exhibit a lower growth and proliferative potential. (E) Imp, together with Chinmo, Lin-28 and Myc, compose a self-renewing module in early larval NBs. Syncrip and E93 are part of an antagonistic module that induces a differentiation-permissive state in NBs and exhausts their self-renewing potential. CB, central brain; wt, wild type.

Temporal patterning driven by sequentially expressed transcription factors appears to be a fundamental mechanism for producing neuronal and glial diversity from a single neural stem cell. Remarkably, beyond allowing the stereotypical generation of progeny with distinct fates over time (Fig. 1), temporal patterning is also used to regulate different aspects of neural progenitor proliferation as these progress throughout development. For example, during embryogenesis, temporal patterning instructs different modes of GMC proliferation. GMCs typically undergo one division to generate two post-mitotic progeny (neurons or glia). However, in some lineages, temporal patterning can trigger transient switches in their proliferation mode, leading them to directly differentiate into a neuron or glia without dividing (Fig. 2A). This effect is mediated by cell-cycle regulators, such as Dacapo (Dap) (a Cyclin-dependent kinase inhibitor in the CIP/KIP family), the expression of which is regulated by embryonic tTFs that are inherited in the GMC (Baumgardt et al., 2014; Ulvklo et al., 2012). Alternatively, temporal patterning can cooperate with the Notch signaling pathway to program or prevent the death of one of the two post-mitotic progeny born during a specific temporal window (Fig. 2A) (Bertet et al., 2014). Temporal patterning can also schedule NB entry into quiescence during the embryonic-to-larval transition by silencing cell-cycle genes [e.g. *E2F transcription factor 1* (*E2f1*), *Cyclin E* and *string*/*cdc25*] (Bahrampour et al., 2017; Baumgardt et al., 2014). Recently, it has been proposed that the sequential expression of two proneural transcription factors, Asense (Ase) and Atonal (Ato), can also schedule an NB asymmetric-to-symmetric division switch, allowing a transient amplification of the NB pool in some regions of the larval brain (Fig. 2B) (Mora et al., 2018).

Although tTFs are transiently expressed, they can induce permanent transcriptional switches in aging NBs. For example, Castor (Cas) can permanently silence the Sox family transcription factor Dichaete (D), while permanently activating Grainyhead (Grh) (Maurange et al., 2008) (Fig. 2C). The sequential permanent transcriptional switches induced by embryonic Cas (D-to-Grh) and early larval Seven up (Svp) [IGF-II mRNA-binding protein (Imp)-to-Syncrip] progressively establish the competence for a subset of NBs, in the abdominal region of the ventral nerve cord, to undergo apoptosis during larval stages upon expression of the Hox gene *abdominal-A* (*abd-A*) (Bello et al., 2003; Maurange et al., 2008) (Fig. 2C).

The remaining NBs exhibit a limited self-renewing potential and undergo cell-cycle exit and differentiation during metamorphosis (Maurange et al., 2008; Truman and Bate, 1988). Therefore, neurogenesis in adults is very sparse, and, if any, does not rely on NB-like cells (Fernández-Hernández et al., 2013). The Svp-mediated Imp-to-Syncrip switch is necessary to terminate NB divisions before adulthood (Genovese et al., 2019; Yang et al., 2017). Early larval NBs express Imp, which promotes a default self-renewing state (Genovese et al., 2019; Samuels et al., 2020). Late larval NBs express Syncrip, which installs a differentiation-permissive state and drives the progressive exhaustion of NB self-renewing potential (Fig. 2D) (Genovese et al., 2019; Yang et al., 2017). The Imp-to-Syncrip switch is scheduled by the sequential expression of Cas and Svp in early larval NBs (Ren et al., 2017; Syed et al., 2017). This switch always operates around the mid-larval stage but is slightly asynchronous in the various NBs denoting that temporal patterning progresses at different speeds in each NB. Upon genetic manipulations, NBs that fail to undergo the Imp-to-Syncrip transition because of temporal patterning defects, do not stop dividing during metamorphosis and remain able to continue dividing in adults (Genovese et al., 2019; Yang et al., 2017). For example, NBs that are mutant for *svp* or that aberrantly express *cas* fail to activate *Syncrip* (Ren et al., 2017; Syed et al., 2017). As such, they retain characteristics of early larval NBs and remain mitotically active in the adult brain (Fig. 2D), thus producing an excess of neurons with an early larval temporal identity (Genovese et al., 2019; Maurange et al., 2008; Yang et al., 2017). This phenotype is also observed in NBs in which *Syncrip* is knocked down (Fig. 2D).

How do Imp and Syncrip respectively promote and restrict NB self-renewal? These two RNA-binding proteins have many RNA targets. Among them, Imp can bind to *Myc* and *chinmo* mRNAs to post-transcriptionally promote their expression (Genovese et al., 2019; Samuels et al., 2020). Myc is a well-known, evolutionarily conserved proto-oncogene that sustains cellular growth and proliferation. Chinmo is a transcription factor of the zinc finger BTB protein family with no clear ortholog identified in mammals yet. Like Myc, Chinmo is a potent proto-oncogene that promotes NB growth and is required for the long-term self-renewing potential of Imp^+^ NBs (Genovese et al., 2019). Lin-28 is another evolutionarily conserved RNA-binding protein and proto-oncogene that is co-expressed with Imp in early larval NBs (Narbonne-Reveau et al., 2016; Syed et al., 2017). Together, Imp, Chinmo, Myc and possibly Lin-28 form an early self-renewing module that is active in early larval NBs, and needs to be silenced in late larval NBs, upon Syncrip expression, to ensure that NBs progressively exhaust their self-renewing potential (Fig. 2D). Whereas Imp promotes *chinmo* mRNA translation, Syncrip somehow blocks it (Genovese et al., 2019). However, the molecular mode of action of Syncrip in NBs remains unclear. In addition to restricting self-renewal, the Imp-to-Syncrip transition also makes NBs competent to differentiate during metamorphosis in response to high levels of ecdysone (Yang et al., 2017). The zinc finger BTB transcription factor Broad (Br) and the ligand-dependent nuclear receptor corepressor-like protein Ecdysone-induced protein 93F (Eip93F; herein referred to as E93) are expressed in late larval NBs during the Syncrip^+^ temporal window (Maurange et al., 2008; Syed et al., 2017). Given the role of Br and E93 in establishing a pro-differentiation chromatin landscape in other tissues, it is likely that they contribute to timing the termination of NB divisions during metamorphosis (Pahl et al., 2019).

In conclusion, a considerable amount of work in *Drosophila* has shown that temporal patterning is a versatile system in neural progenitors. Temporal patterning generates sequences of competence windows endowing precursors with specific proliferative and differentiation potentials. Thus, by coordinating fate with self-renewing abilities, temporal patterning allows for an exquisite regulation of the numbers of each neuron and glial type that will constitute a final lineage.

## Temporal patterning and the specification of tumor-prone cells

**Fig. 3.**
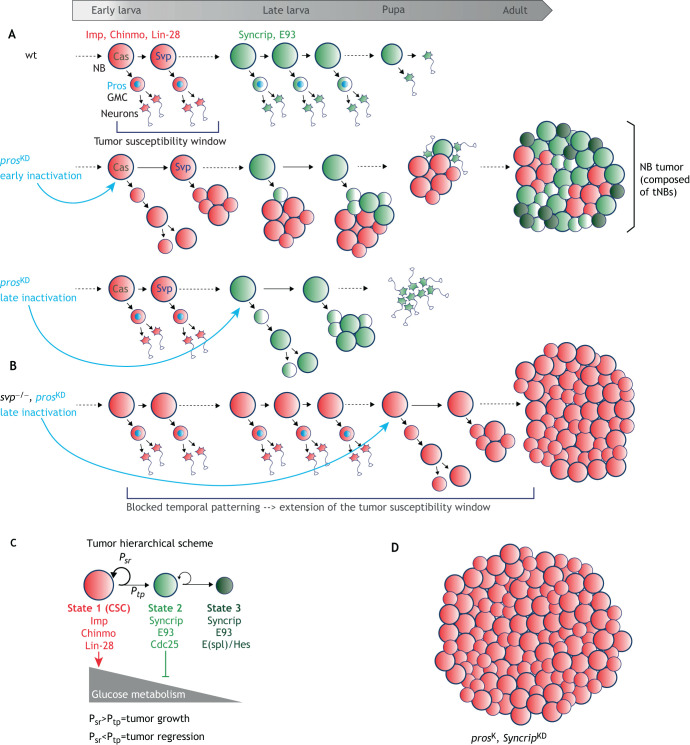
**Co-option of larval temporal patterning drives** **cellular hierarchy in neurodevelopmental tumors.** (A) Early larval NBs (red) express *chinmo*, *Imp* and *lin-28*; late larval NBs (green) express *Syncrip* and *E93*. Pros (light blue) is present in GMCs to promote their differentiation after one division. Early knockdown (KD) of Pros in the NB lineage causes NB tumors. Tumors continue growing in adults and are composed of a mix of NB-like cells (tNBs) that transcriptionally resemble early larval Imp^+^ NBs and late larval Syncrip^+^ NBs. Late KD of Pros from Syncrip^+^ NBs triggers transient NB amplification but supernumerary NBs remain competent to differentiate during metamorphosis like wild-type (wt) NBs, leading to an absence of tumors in adults. Thus, the early Imp^+^ window is tumor prone. (B) Stalling temporal patterning progression in NBs (*svp^−^*) delays or prevents the Imp-to-Syncrip temporal transition, leading to an extension of the tumor-susceptible window. In this context, late inactivation of *pros* can lead to tumorigenesis. (C) NB tumors are hierarchical. Imp^+^Chinmo^+^Lin-28^+^ tNBs (State 1) behave like CSCs. They can self-renew or progress through temporal patterning to give rise to Syncrip^+^E93^+^ tNBs (State 2). Syncrip^+^E93^+^ tNBs act as transient amplifying progenitors and are prone to exiting the cell cycle (State 3). If Imp^+^ tNBs have a probability to self-renew (P_sr_) that is higher than the probability to undergo temporal progression (P_tp_), the Imp^+^ tNBs can perpetuate themselves, leading to tumor growth. If P_sr_ becomes lower than P_tp_, then Imp^+^ tNBs are progressively eliminated and the tumor regresses. (D) Inactivation of both *pros* and *Syncrip* leads to tumors only composed of Imp^+^ tNBs, which exhibit a higher growth rate.

The deterministic progression of temporal patterning driven by cross-regulatory interactions between tTFs in *Drosophila* NBs can act as a counting mechanism to limit self-renewal during development, thus ensuring the termination of cell division before adulthood. However, a large body of work has shown that NB lineages in the making are particularly prone to tumorigenic overproliferation. For example, inactivation of the transcription factor Prospero (Pros) in type I NBs prevents the proper differentiation of their GMCs and the production of post-mitotic progeny. Instead, GMCs revert to NB-like cells that will continue to divide, leading to the exponential amplification of cells with NB-like properties (Bello et al., 2006; Betschinger et al., 2006; Choksi et al., 2006). If *pros* is inactivated in early Imp^+^ larval NBs (also expressing *chinmo* and *lin-28*), a subset of the supernumerary NB-like cells fails to terminate divisions during metamorphosis and continues to proliferate into adulthood (Fig. 3A), rapidly generating large tumors that ultimately kill the fly (Narbonne-Reveau et al., 2016). Such tumors can be transplanted in the abdomen of adult flies for years without losing their ability to grow and populate adjacent tissues (Caussinus and Gonzalez, 2005). In contrast, inactivation of *pros* in late Syncrip^+^ NBs and their GMCs triggers transient NB-like amplification but fails to generate tumors in the adults (Fig. 3A). Thus, the Imp^+^ temporal window constitutes an early developmental window of cancer susceptibility and Imp appears to prime cells for tumorigenic self-renewal. Blocking temporal progression in Imp^+^ NBs (by inactivating *svp* in early larval NBs, for example) extends the cancer susceptibility window by preventing the Imp-to-Syncrip switch (Fig. 3B) (Narbonne-Reveau et al., 2016). Interestingly, the inactivation of *Snf5-related 1* (*Snr1*), the *Drosophila* ortholog of human *SMARCB1* (inactivation of which causes ATRTs), in type II NBs also causes aggressive tumors, showing that the tumor-suppressive activity of *Snr1/SMARCB1* in neural progenitors is conserved throughout evolution (Eroglu et al., 2014; Koe et al., 2014). The inactivation of other genes involved in NB asymmetric divisions (*miranda*), in the maturation of InPs (e.g. *brat*, *numb*, *earmuff*), or in the maturation of neurons (*nerfin-1*) can also induce NB overproliferation and tumor growth (Froldi et al., 2015; Hakes and Brand, 2019). In most, if not all cases, the cells of origins of NB tumors are Imp^+^ early larval NBs or their daughter cells (GMCs or InPs), or Imp^+^ maturing neurons (Narbonne-Reveau et al., 2016). Thus, more than the cell type, it is the temporal identity of the cell of origin that determines its malignant susceptibility.

Mutations or genetic manipulations that lead to Notch signaling overactivity in the InPs of type II NBs lead to their tumorigenic amplification (Bowman et al., 2008). InPs typically do up to six asymmetric divisions and progress through the D→Grh→Ey temporal patterning system (Bayraktar and Doe, 2013) (Fig. 1C and Box 1). However, Ey seems to terminate a window of tumor susceptibility as late Ey^+^ InPs appear refractory to tumor transformation induced by Notch overactivity (Farnsworth et al., 2015). Again, silencing this late component of InP temporal patterning can extend the window of tumor susceptibility.

These studies demonstrate that intrinsic temporal programs in *Drosophila* neural stem cells can delineate specific windows of malignant susceptibility. Moreover, this work establishes *Drosophila* NB tumors as a relevant model to investigate neural tumors with developmental origins and raises the possibility that similar temporal mechanisms specify tumor-prone cells at the origin of childhood CNS cancers.

## Co-opted temporal patterning and the regulation of cellular hierarchy and metabolic heterogeneity in tumors

The understanding of the mechanisms underlying the growth of *Drosophila* NB tumors recently progressed further thanks to the use of single-cell RNA sequencing (RNA-seq) approaches. Single-cell RNA-seq studies are revolutionizing the field of developmental and cancer biology, as they allow a more systematic description of the cellular heterogeneity present within a tissue (Pennisi, 2018). Moreover, specific algorithms can be used to infer transcriptional trajectories operating within a cell population, reflecting cells progressing through distinct transcriptional states (Tritschler et al., 2019). When applied to single-cell transcriptomes of NB tumors, such algorithms, known as pseudotime ordering, reveal that NB-like cells composing tumors (hereafter referred to as tNBs) follow trajectories recapitulating parts of larval temporal patterning (Genovese et al., 2019). Together with *in vivo* lineage analysis and numerical simulation, this study has demonstrated that NB tumors in adult flies, induced by *pros* inactivation during early larval stages, are mainly composed of tNBs progressing through three distinct temporal states that follow a fine-tuned hierarchical scheme (Fig. 3C). A ‘State 1’ subpopulation of tNBs expresses Imp and other typical markers associated with early larval NBs (Chinmo, Lin-28, etc). Imp^+^ tNBs continuously divide to self-renew, perpetuating themselves and propagating tumor growth. However, with a low probability, Imp^+^ tNBs are able to undergo the Imp-to-Syncrip temporal transition, giving rise to Syncrip^+^ tNBs. The latter also express the transcription factor E93, a marker for differentiation-prone NBs during development (State 2). As such, ‘State 2’ Syncrip^+^ E93^+^ tNBs resemble late L3 larval NBs. Syncrip^+^ E93^+^ tNBs can divide to self-renew but they have a strong tendency to exit the cell cycle and overexpress Notch target genes of the E(spl)/Hes family (State 3). It is unclear whether ‘State 3’ represents an ultimate temporal state during larval or pupal development, because late Syncrip^+^ E93^+^ NBs differentiate during metamorphosis before exiting the cell cycle, owing to a pupal burst of ecdysone (Homem et al., 2014). However, E(spl)/Hes genes are activated in late-embryonic NBs to silence cell-cycle genes (Bivik et al., 2016), and they also counteract neuronal differentiation by favoring quiescent neural stem cell states in vertebrates (Sueda et al., 2019), suggesting that they could be important to induce and maintain an inactive NB population in tumors. In tumors, as in development, Syncrip^+^ tNBs do not revert to the Imp^+^ state. Thus, NB tumors are hierarchical and Imp^+^Chinmo^+^Lin-28^+^ tNBs exhibit the characteristics of cancer stem cells (CSCs) as they can both perpetuate tumor growth and promote cellular heterogeneity (Nassar and Blanpain, 2016). Whereas Imp functions in a positive-feedback loop with Chinmo to sustain the CSC population (Narbonne-Reveau et al., 2016), Syncrip silences the Chinmo/Imp module to suppress the CSC state, therefore acting as a tumor suppressor (Genovese et al., 2019). Consequently, inactivation of Syncrip dramatically boosts the CSC population and tumor growth (Fig. 3D). Thus, Imp and Syncrip have an antagonistic role in controlling the CSC population. In this context, CSC properties are conferred by the proto-oncogenic/self-renewing module – composed of Imp, Chinmo, Lin-28 and Myc – that was active in the cell of origin (an early larval GMC).

How does Syncrip restrict tNB self-renewing abilities? Interestingly, Syncrip was found to trigger the downregulation of the expression of metabolic genes (glycolysis and oxidative phosphorylation pathways) (Genovese et al., 2019). This suggests that the tendency of Syncrip^+^ tNBs to rapidly exit the cell cycle could be a consequence of metabolic exhaustion. This study demonstrates that co-option of the larval temporal patterning program during early developmental stages induces hierarchical tumors and provides a tumor-intrinsic mechanism that creates metabolic heterogeneity to control the proliferative potential of the various tumor cells. It also uncovers a central role for RNA-binding proteins in governing tumor cell hierarchy.

Whereas the Imp-to-Syncrip switch is systematic in NBs during larval development (promoted by tTF progression), which leads to an absence of Imp^+^ NBs in late larvae, this transition seems to be regulated in a more stochastic manner in the tumor context, which allows maintenance of the Imp^+^ CSC-like population of tNBs (Genovese et al., 2019). However, the molecular principles underlying this stochastic regulation are unknown. Unravelling the mechanisms controlling the Imp-to-Syncrip switch in the tumor context could provide new perspectives to understand how CSC populations are regulated in tumors.

## Evidence of temporal patterning in mammalian neural progenitors

Could co-option of temporal patterning in neural progenitors provide a new conceptual framework to understand how pediatric CNS cancers are induced and progress in humans? Until recently, the evidence of temporal patterning in mammalian neural progenitors was sparse (Jacob et al., 2008; Kohwi and Doe, 2013), largely because of the inability to easily capture dynamic transcriptional profiles of single neural progenitors over time. However, accumulating evidence now suggests that many neural stem cells undergo stereotyped temporal patterns of proliferative, neurogenic and gliogenic divisions during early development, which are controlled by a combination of stem cell-encoded programs and extrinsic cues. In particular, the advance of single-cell transcriptomic technologies now allows for the reconstruction of temporal trajectories in neural progenitors and the identification of transcriptional programs governing neural stem cell proliferative and neurogenic potentials.

**Fig. 4.**
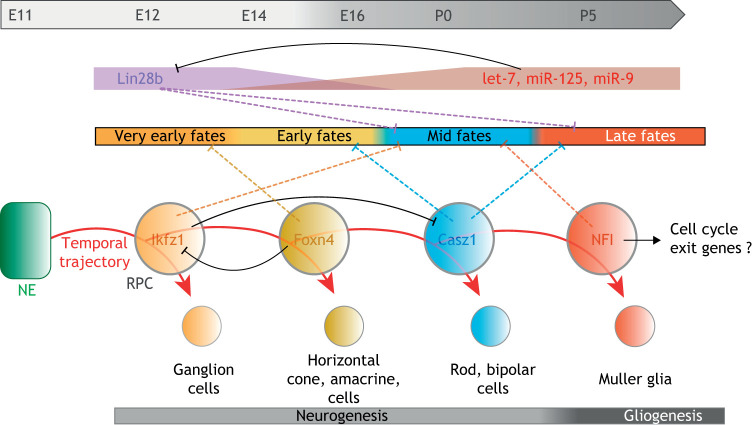
**Temporal patterning in mouse retinal progenitors.** Retinal progenitor cells (RPCs) are converted from a neuroepithelium (NE) during embryonic stages. Together with genetic experiments, recapitulation of transcriptional trajectories by single-cell RNA-seq suggests that fetal RPCs transit throughout various competence windows that bias the production of various neuronal and glial fates. A series of tTFs (Ikfz1→Foxn4→Casz1→NFI) is emerging to drive progression throughout these competence windows. NFI coordinates the last glial fate with cell-cycle exit, thereby terminating the lineage. tTF progression is paralleled in RPCs with a gradient of microRNAs that terminate the early competence to generate ganglion cells by silencing *Lin28b*. It is unclear whether the microRNA gradient and the tTF series cross-regulate or constitute independent temporal systems. E, embryonic day; P, postnatal day.

The developing retina provides to date one of the most striking examples of temporal patterning in mammalian neural progenitors. Retinal progenitor cells (RPCs) are multipotent neural progenitor cells that generate various types of neurons and glia in a stereotypic sequence (Cepko, 2014). Pioneer studies have shown that the genes Ikaros family zinc finger 1 (*Ikzf1*) and zinc finger protein castor homolog 1 (*Casz1*), respective orthologs of *Drosophila hb* and *cas*, are sequentially expressed and cross-regulate in mouse RPCs to bias the generation of early (ganglion, horizontal and amacrine cells) and mid-late (rod, bipolar cells) cell types, respectively (Elliott et al., 2008; Konstantinides et al., 2015; Mattar et al., 2015). This suggests a striking conservation of the Hb→Cas temporal module from insects to mammalian neural progenitors. More recently, single-cell transcriptomics has helped to identify two novel factors in this series. The transcription factor forkhead box N4 (Foxn4) represses the fate of ganglion cells, which constitute the earliest type of neurons being generated in the retina, while promoting subsequent fates (Liu et al., 2020). In addition, genes of the Nuclear factor I (NFI) family (*Nfia*, *Nfib* and *Nfix*) specify both the fate of late-born progeny (Müller glia) and trigger the cell-cycle exit of RPCs (Clark et al., 2019). Cross-regulatory interactions further strengthen the hypothesis of an Ikfz1→Foxn4→Casz1→NFI transcription factor-driven temporal series that biases the sequential production of different neuronal and glial fates as RPCs divide along fetal development (Fig. 4). Similar to temporal patterning in type I NBs (Maurange et al., 2008), this temporal patterning system coordinates progression throughout various competence windows with termination of proliferation (Fig. 4). Pseudotime ordering and machine-learning strategies of these single-cell data also revealed lists of more complex patterns of co-regulated genes expressed in early and late RPCs (Clark et al., 2019). Lastly, evidence that Casz1 suppresses late gliogenesis by interacting with chromatin-modifying complexes provides a mechanism by which tTFs establish competence windows (Mattar et al., 2020 preprint).

Interestingly, the Ikfz1→Foxn4→Casz1→NFI temporal series appears to be paralleled with a temporal gradient of microRNAs (let-7, miR-125 and miR-9) that progressively terminates the competence window for generating ganglion cells (Fig. 4). These microRNAs partly act by silencing the lin-28 homolog B (*Lin28b*) Lin28b (La Torre et al., 2013). Thus, Lin-28 RNA-binding proteins label and favor early temporal identities in *Drosophila* larval NBs and mouse RPCs. However, unlike tTFs and RNA-binding proteins in *Drosophila* NBs, it is unclear whether the Ikfz1→Foxn4→Casz1→NFI tTF series and the Lin28b/microRNA gradients in mouse RPCs cross-regulate or represent independent transcriptionally and post-transcriptionally controlled temporal systems.

In other regions of the mammalian brain, temporal patterning is being molecularly dissected. Cortical progenitors, known as apical progenitors (APs), also undergo a sequence of events from proliferative symmetric divisions to neurogenic asymmetric divisions (during which six layers of neurons are produced), to gliogenic divisions and terminal differentiation (Greig et al., 2013). The Svp orthologs COUP transcription factor 1 (NR2F1) and COUP transcription factor 2 (NR2F2) have been proposed to promote a temporal switch, allowing APs to switch from generating early types of neurons (deep neuronal layers) to late types of neurons (superficial neuronal layers) and gliogenesis (Faedo et al., 2008; Naka et al., 2008). Moreover, NR2F1 has been shown to limit neural progenitor self-renewal (Bertacchi et al., 2020). Single-cell transcriptomics have now identified several transcriptional switches operating in mouse APs along embryonic development. Some of them appear to promote the early proliferative to neurogenic-division switch, whereas others are likely involved in establishing transcriptional profiles that are transmitted to neuronal progeny, specifying deep or upper-layer neuronal fates (Telley et al., 2019). Temporal progression through the transcriptional states that specify deep and upper-layer neuronal fates is largely independent of cell-cycle progression but is less efficient in cultured isolated cells (Okamoto et al., 2016). This suggests that temporal transitions in cortical APs are governed by both cell-intrinsic and -extrinsic mechanisms. Although the mechanisms of temporal progression are unclear, early temporal identity in APs [embryonic day (E)12-E13] was associated with high expression of cell-cycle regulators, DNA replication, transcriptional and chromatin regulators (Telley et al., 2019). Reminiscent of *Drosophila* early larval NBs, mouse Imp1/Igf2bp1 and Imp2/Igf2bp2, as well as Lin28a, are strongly expressed in early APs and promote self-renewal (Nishino et al., 2013; Yang et al., 2015). The transcriptome of late APs found at E14-E15 indicates an over-representation of genes involved in ion transport, cell-cell and cell-matrix interactions, suggesting that late APs may be more capable of sensing their environment than early APs. Finally, glia-related genes become activated at this stage, thus foreshadowing the later neurogenic-to-gliogenic transition (Telley et al., 2019).

**Fig. 5.**
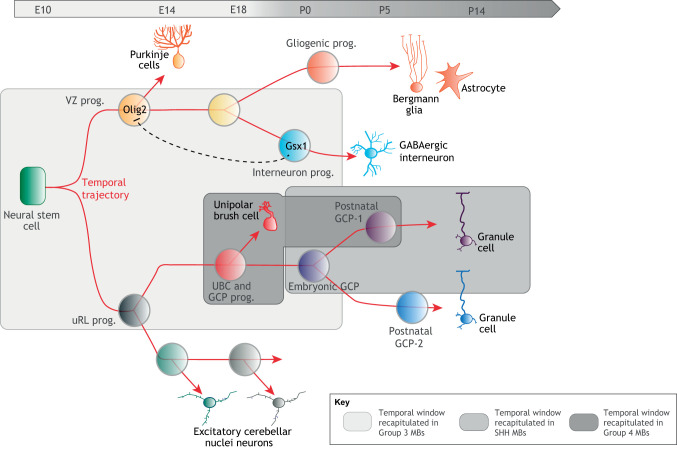
**Transcriptional trajectory during development and tumorigenesis in the cerebellum.** Single-cell RNA-seq of the developing murine cerebellum reveals the transcriptional trajectory along the different branches of the cerebellar lineage. As the developmental program unfolds, progenitors progress throughout various competence states and produce diverse types of progeny. Olig2 and Gsx1 have been proposed to regulate temporal identities in VZ progenitors, suggesting that multiple temporal patterning systems may concurrently progress in the various branches of the lineage to regulate progenitor competence and proliferative properties. Specific subparts of the transcriptional trajectory are recapitulated in Group 3, Group 4 and SHH MBs. An attractive hypothesis is that distinct temporal patterning systems possibly coopted from different cells of origin may govern many aspects of tumor properties in the different MB subgroups. GCP, granule cell precursor; MB, medulloblastoma; prog., progenitor; UBC, unipolar brush cell; uRL, upper rhombic lip; VZ, ventricular zone.

The cerebellum contains half of the neurons in the adult human brain. This structure emanates from a neuroepithelium called the rhombic lip. Previous genetic and lineage tracing studies have proposed that cerebellar progenitors progress through various temporal identities to produce specific neuronal subtypes at different developmental times. For example, embryonic ventricular zone (VZ) progenitors sequentially express the transcription factors oligodendrocyte transcription factor 2 (Olig2) and GS homeobox 1 (Gsx1), allowing the sequential production of Purkinje cells and GABAergic neurons (Seto et al., 2014) (Fig. 5). This suggests the existence of a temporal patterning system in the VZ sublineage. Recently, single-cell RNA-seq applied during the course of murine cerebellar development has highlighted developmental trajectories and complex transcriptional cascades possibly indicative of multiple temporal patterning systems (Carter et al., 2018; Wizeman et al., 2019). Interestingly, during the course of lineage progression, granule cell precursors (GCPs) undergo a formidable phase of expansion to generate the large number of granule cells responsible for the massive foliation of the cerebellar surface of the adult cerebellum in mammals. Whether the competence to amplify at this stage of lineage progression is scheduled by a temporal patterning system remains to be investigated. The unanticipated complexity of cerebellar development revealed by single-cell RNA-seq analysis leaves open the possibility that multiple temporal programs are concurrently activated in the various branches of the cerebellar lineage to specify the identities and numbers of the different types of progeny (Fig. 5).

Lastly, single-cell transcriptomics have also revealed neuronal fates specified by the order in which cells arise in the developing spinal cord (Delile et al., 2019; Sagner and Briscoe, 2019). In the mouse embryonic cervical domain, three layers of neurons are produced sequentially and can be labeled with different combinations of transcription factors. As in the retina, NFI transcription factors (Nfia, Nfib) specify late-born fates, possibly suggesting a general role for NFI factors in terminating temporal cascades among some neural lineages (Delile et al., 2019). Interestingly, NFI factors are also required to initiate the neurogenic-to-gliogenic switch in spinal cord progenitors (Deneen et al., 2006), therefore possibly linking the neurogenic-to-gliogenic transition to the end of the neuronal temporal specification program. However, how temporal specification of spinal cord neurons is established remains to be deciphered.

Such studies are revealing that neural stem cell-encoded temporal patterning systems are probably widely used during the development of the different regions of the mammalian CNS to coordinate the mode of division and self-renewing potential of progenitors with developmental competence. This leads to the production of large repertoires of neurons and glia in defined numbers. The combination of single-cell RNA-seq technologies associated with machine-learning strategies pave the way for a comprehensive exploration of the temporal patterning programs deployed in different types of neural progenitors and their progeny. Further refinements of transcriptional dynamics in progenitors supported by genetic studies will be necessary to identify the core temporal patterns that define various competence windows and the underlying cell-intrinsic and -extrinsic mechanisms that promote temporal transitions. A future challenge will be to investigate temporal patterning mechanisms during human brain development and explore how their deregulation could underlie neurodevelopmental disorders (Schafer et al., 2019).

## Recapitulation of temporal patterning programs in CNS pediatric tumors?

Since temporal patterning in mammalian neural progenitors remains poorly explored, it is still hypothetical whether its co-option is involved in the etiology of human childhood malignancies. However, several observations suggest an important role. First, the fact that many pediatric CNS tumors can only be induced during very specific windows of development argues for the existence of temporal mechanisms regulating transient tumor-prone cellular states (Han et al., 2016; Pathania et al., 2017; Singh et al., 2018). Second, as shown below, the comparison of single-cell transcriptomes from human and mouse developing brains with various pediatric CNS tumors supports the hypothesis that partial recapitulation of fetal transcription programs is a characteristic of CNS pediatric tumors.

The most spectacular example so far probably arises from recent studies of cerebellar tumors, known as medulloblastoma, which constitute the most frequent CNS tumors in children. They can be divided into four subgroups – WNT, SHH, Group 3 and Group 4 – presumably originating from different cells upon various genetic insults (Northcott et al., 2017). Pseudotime reconstruction of single-cell RNA-seq data from human medulloblastomas identified specific transcriptional trajectories for each subgroup, recapitulating subparts of the temporal trajectories observed during lineage progression in the developing cerebellum. In particular, most medulloblastoma appears to be locked in a specific developmental/temporal window (Hovestadt et al., 2019; Vladoiu et al., 2019) (Fig. 5). For example, SHH medulloblastomas recapitulate temporal progression throughout the prolonged clonal expansion of GCPs occurring during fetal and early postnatal stages. Interestingly, these different temporal stages of GCP differentiation elicit distinct responses to therapeutic treatments (Ocasio et al., 2019), suggesting that knowledge on the co-opted temporal patterning window could help to inform the therapeutic strategy.

Group 3 medulloblastomas contain different types of progenitors from the VZ and upper rhombic lip (uRL) sublineages, suggesting a cell of origin with an early temporal identity possibly associated with multipotency and high self-renewing potential. In contrast, Group 4 medulloblastomas appear to be composed of more-differentiated progenitors along the uRL lineage, mostly reminiscent of unipolar brush cell progenitors, and more-differentiated progeny (Hovestadt et al., 2019; Vladoiu et al., 2019) (Fig. 5). The WNT group recapitulates a developmental trajectory matching the mossy fiber neuron lineage generated in the dorsal brain stem during fetal stages (Jessa et al., 2019). Interestingly, Groups 3 and 4 highly express LIN28B (Hovestadt et al., 2014), while the WNT group sometimes exhibits inactivating mutations in the *SYNCRIP* gene (Northcott et al., 2017). Overexpression of *LIN28B* and inactivation of *SYNCRIP*, orthologs of two members of the temporal gene network in *Drosophila*, are therefore oncogenic events conserved in CNS developmental tumors from insects to humans. An attractive hypothesis is that distinct temporal patterning systems co-opted from different cells of origin may govern many aspects of tumor properties, including cellular hierarchies, in the different medulloblastoma subgroups.

Other pediatric CNS tumors also appear to partially recapitulate the fetal development of some specific neural lineages, such as embryonal tumors with multilayered rosettes or pediatric high-grade gliomas (Jessa et al., 2019; Pathania et al., 2017).

Together, these single-cell RNA-seq studies demonstrate that CNS childhood tumors consistently recapitulate portions of lineage-specific fetal developmental programs and that such knowledge can be used to effectively classify the tumor subtype and inform treatment to target cells at the apex of the tumor hierarchy.

## Conclusions and perspectives

The exploration of temporal patterning in mammals is still in its infancy, but the underlying concepts uncovered in *Drosophila* appear largely transposable, with sometimes conserved molecular players (Table 1). Single-cell RNA-seq studies are revealing complex transcriptional trajectories for different types of neural progenitors with increasing accuracy. Genetic studies are now required to understand how these transcriptional trajectories are deployed in neural progenitors, and how the underlying temporal gene network generates temporal windows and competence states to coordinate the production of various fates with a specific proliferation potential along a neural lineage. Does this process essentially result from cross-regulatory interactions between series of transcription factors (as for *Drosophila*), or is it mainly driven by extrinsic signals? More likely, a combination of both could account for the stochasticity in cell-fate decisions that has been observed in the retina or cerebral cortex, which contributes to both more-robust patterning and flexibility for the generation of novel region-specific lineages throughout evolution (Llorca et al., 2019; Mattar et al., 2015).

**Table 1.**
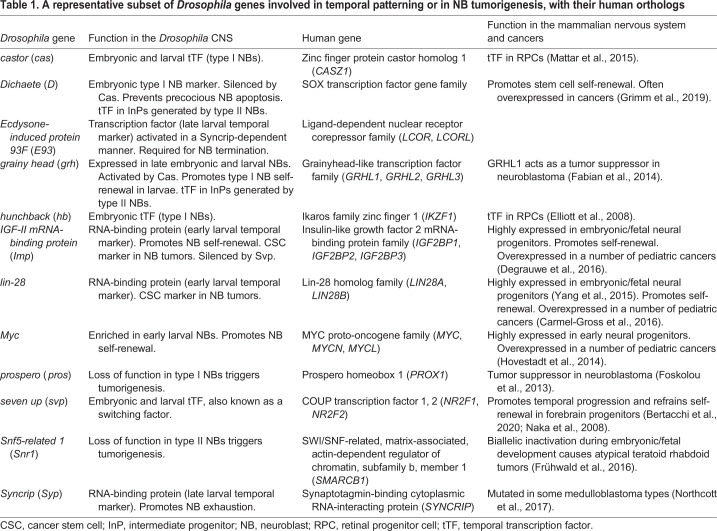
**A representative subset of *Drosophila* genes involved in temporal patterning or in NB tumorigenesis, with their human orthologs**

Beyond the CNS, the concept of temporal patterning may apply to other tissues, such as the hematopoietic or neural crest cell lineages. Fetal hematopoietic stem cells and embryonic neural crest cells generate large and complex lineages that are also susceptible to malignant transformation during embryonic and fetal stages, giving rise to leukemias and neuroblastoma, respectively (Marshall et al., 2014).

Although the partial recapitulation of temporal programs appears to be a common theme in CNS childhood tumors, it remains largely unexplored how they control tumor hierarchy, cellular organization and response to therapeutic treatment. Work on *Drosophila* has been instrumental in understanding how an early window of the temporal program operating in neural progenitors can be co-opted to drive tumorigenesis and establish a cellular hierarchy. In this context, the temporal hierarchy during development prefigures the tumor hierarchy, and CSCs are defined by a proto-oncogenic module already active in the cell of origin. It is likely that many characteristics attributed to CSCs (such as self-renewal, resistance to cell death and differentiation, chemoresistance) are developmental traits. The characterization of temporal patterning programs occurring in the various mammalian neural progenitors could therefore help with predicting the gene network defining CSCs and response to treatments.

During development, temporal patterning programs systematically progress in all neural progenitors to ultimately ensure lineage termination. In contrast, in pediatric tumors, temporal progression seems incomplete, and a subset of progenitors appears stalled in an early temporal state, perpetuating proliferation. In *Drosophila* NB tumors, temporal transitions are altered, shifting from a systematic-to-stochastic occurrence, locking neural lineages into ‘perpetual development’. Understanding how the mechanisms regulating temporal transitions are reconfigured during tumorigenesis could open new therapeutic perspectives aiming at forcing temporal progression and the subsequent exhaustion of the CSC pool.
